# Transitions in Functional Status and Frailty Among Post-Hospitalized COVID-19 Oldest-Old Adults: An 18-Month Follow-Up Study

**DOI:** 10.3390/jcm15010209

**Published:** 2025-12-27

**Authors:** Rafael Saavedra-Palacios, Alejandro Álvarez-Bustos, Lucía Furones-Fernández, Ángel Chen-Chim, María Teresa Boimorto-Medina, Rebeca Martiño-Córdoba, Pedro Gil-Gregorio, Walter Sepúlveda-Loyola, Isabel Rodríguez-Sánchez

**Affiliations:** 1 Geriatrics Department, Hospital del Río, Av. 24 de Mayo, Cuenca 010150, Ecuador; 2 Department of Health, Universidad Villanueva, 28034 Madrid, Spain; a.alvarezbu@gmail.com; 3 Geriatrics Department, Hospital Universitario Infanta Elena, Av. De los Reyes Católicos, 21, Valdemoro, 28342 Madrid, Spain; 4 Geriatrics Department, Hôpitaux Robert Schuman, 9 Rue Edward Steichen, Neudorf-Weimershof, 2540 Luxembourg, Luxembourg; 5 Geriatrics Department, Hospital General de Villalba, Carretera M-608, Km 41, Collado Villalba, 28400 Madrid, Spain; 6 Geriatrics Medicine Department, Hospital Universitario Clínico San Carlos, C/Profesor Martín Lagos s/n, 28040 Madrid, Spain; 7 Faculty of Health and Social Sciences, Universidad de Las Américas, Santiago de Chile 8370040, Chile; 8 Center for Research in Biological and Chemical Sciences, Universidad de Las Américas, Santiago de Chile 8370040, Chile; 9 Instituto de Investigación Sanitaria del Hospital Clínico San Carlos, Fundación para la Investigación Biomédica (IdISCC), 28040 Madrid, Spain

**Keywords:** SARS-CoV-2, functional decline, physical decline, Barthel index, length of stay

## Abstract

**Objectives**: This study seeks to analyze physical function and frailty transitions in older adults at 1, 12, and 18 months after hospital discharge for coronavirus disease (COVID-19). Furthermore, we examined risk factors associated with patterns of changes. **Methods**: This is an observational, longitudinal, unicentric study. Participants aged ≥80 years who were admitted to a Spanish tertiary hospital and survived COVID-19 were included. Functional status was evaluated at baseline and after 1, 12, and 18 months of discharge using the Barthel Index (BI) and Functional Ambulation Category (FAC), and frailty was assessed using the FRAIL scale. Clinical, sociodemographic, and functional parameters were evaluated as covariates. **Results**: Two hundred and thirty-three individuals (mean age 86.2 ± 4.0 years, 48.9% men) were included. Patterns of changes were classified into three categories as follows: no changes (i.e., same score at baseline and follow-up), improvement (i.e., better results at follow-up), and worsening (i.e., detriments at follow-up). Worse frailty status at baseline [relative risk ratio (RRR) = 1.39] and longer length of stay (RRR = 1.03) were associated with higher risk of frailty impairment (worsening pattern). Older age (RRR = 1.11), longer length of stay (RRR = 1.03), and worse baseline physical function (RRR = 1.01) and FRAIL scores (RRR = 1.37) were associated with impairments in physical status at 18 months. **Conclusions**: Three distinct patterns of changes in functionality and frailty were observed among older adults discharged after COVID-19. Age, length of stay, and worse frailty and functional status at baseline emerged as significant risk factors for worsening in the examined conditions.

## 1. Introduction

The World Health Organization (WHO) estimates that 778 million individuals have been infected by the coronavirus SARS-CoV-2 since 2019, which is responsible for more than 7 million deaths around the world, mainly due to respiratory complications [1,2]. Spain was one of the most affected countries, with 14 million cases and 122,000 deaths confirmed so far [1]. Older adults are particularly vulnerable to SARS-CoV-2 and exhibit the worst prognosis [3].

Indeed, the susceptibility and fatality rates to SARS-CoV-2 increase as individuals age, with results indicating that nearly 90% of the mortality rates occur among older adults [3,4]. Notably, more than 50% of the mortality rates reflect deaths in individuals 80+ years [4], which represents a 5-fold higher mortality risk in comparison to other age groups [3].

Functional problems are a common result of SARS-CoV-2 [5]. For instance, data from the Canadian Longitudinal Study on Aging found that COVID-19 survivors were at a higher risk of worsening mobility and physical function, compared to those not infected [5]. These findings were expanded by Pérez-Rodríguez et al. [6] and Prampart et al. [7], who reported significant physical impairments in 20% of older adults infected by COVID-19, while worsening in frailty status was observed in 27%. This scenario is not easily reversed, as observed in a Spanish study that found that functional decline persisted in nearly 50% of the examined individuals after 6 months of discharge [8,9].

The early identification of those individuals at elevated risk of negative events is critical to promoting prompt interventions, improving prognosis, and reducing disease burden. Numerous candidate variables (e.g., sociodemographic, health, economic, COVID-19 severity) linked to worsening functionality and frailty have been investigated. Results are still scarce for some of them, whereas others remain conflicting. Furthermore, some of the significant risk factors are not routinely examined in clinical practice, which indicates that their evaluation will be restricted to some studies, limiting their assumption as important predictor parameters [10].

Particularly, severe dependence at baseline and after discharge increases the risk of persistent functional deterioration at three months by 24-fold [6,9]. Other risk factors include age, length of hospital stay, comorbidities, biochemical profile, and complications due to COVID-19 [7,11,12,13,14,15]. However, most of these findings refer to young populations followed by short-term periods [16,17,18].

Based on these premises, the present study aimed at analyzing the long-term (18 months) trajectories of physical function and frailty among very old adults discharged after COVID-19 hospitalization. Furthermore, numerous potential risk factors were examined.

## 2. Material and Methods

### 2.1. Study Design and Participants

This is an observational, longitudinal, and monocentric study. The study was conducted according to the principles of the Declaration of Helsinki, and the protocol was approved by the Research Ethical Committee of the Clinical San Carlos Hospital (22/099-E, 18 February 2022). Written informed consent was obtained from all participants or their legal representatives, when necessary, prior to enrollment. The study was conducted in accordance with the Strengthening the Reporting of Observational Studies in Epidemiology (STROBE) guidelines [19].

Data were obtained from patients aged 80 years and older diagnosed with SARS-CoV-2 infection who were consecutively admitted to a university tertiary hospital located in Madrid, Spain, between 4 March 2020 and 16 May 2020. This hospital is one of the biggest in Spain and is located in the city center of Madrid. The hospital comprises more than 5 thousand professionals and 929 beds and is responsible for the healthcare of nearly 400 thousand individuals.

Follow-up visits were performed 1, 12, and 18 months after discharge. Only data of those individuals who had completed data for the entire follow-up period and were diagnosed using a polymerase chain reaction test were analyzed.

### 2.2. Main Outcomes

Functional status was evaluated using two self-reported assessment tools, namely, the Barthel Index (BI) and the Functional Ambulation Category (FAC), while frailty was identified using the FRAIL scale. Follow-up interviews were conducted by one trained geriatrician at 1, 12, and 18 months after baseline data collection through telephone contact with participants or their caregivers, in cases where it was necessary. Functional and frailty trajectories were classified into three groups (no changes, improvement, and worsening) in each follow-up evaluation, in comparison to baseline, according to changes of ≥5 points in the BI [20] and ≥1 in the FAC and FRAIL scales [21,22], regardless of the direction (i.e., increase or decrease).

#### 2.2.1. Barthel Index

The Barthel Index (BI) is a 10-item, self-reported instrument widely used to evaluate an individual’s ability to independently perform activities of daily living (ADL). These activities include feeding, bathing, grooming, dressing, bowel and bladder control, toilet use, transfers (e.g., moving from bed to chair), mobility on level surfaces, and stair climbing [20]. Each item reflects a fundamental domain of functional autonomy, and responses quantify the degree of assistance required for task completion. The BI produces a final score ranging from 0 to 100, with higher scores indicating greater functional independence. This scoring framework allows clinicians and researchers to classify individuals according to their level of dependency. Scores between 90 and 100 denote full or near-full independence, whereas scores of 60 to 85 indicate mild dependency. Individuals with scores in the 30 to 55 range are considered to have moderate dependency, while those scoring below 30 are categorized as having severe dependency. This categorization supports clinical decision making, rehabilitation planning, and the monitoring of functional outcomes over time.

#### 2.2.2. Functional Ambulation Category

The FAC scale assesses the ability to walk and move independently through the examination of the degree of physical support and/or supervision required during ambulation, regardless of the utilization of assistive devices [22]. Results are classified into six categories ranging from 0 (i.e., Nonfunctional Ambulation) to 5 (i.e., Ambulator-Independent), with higher scores indicating greater independence.

#### 2.2.3. FRAIL

Frailty status was identified with the FRAIL scale [21]. This assessment tool evaluates the presence of frailty through the self-reported presence of fatigue, resistance, ambulation, illness, and unintentional weight loss. A point is given for the presence of each condition. Participants who met no criteria are classified as robust, prefrail (1–2 criteria), and frail (≥3 criteria).

### 2.3. Risk Factors

Risk factors included variables from distinct domains: (a) sociodemographic (i.e., age, sex, and setting); (b) comorbidities, using the Charlson index [23] and the Cumulative Illness Rating Scale-Geriatric (CIRS-G) [24]; (c) usual pharmacological therapy, which was classified into three groups, including no polypharmacy (<5 drugs), polypharmacy (5–9 drugs), and hyperpolypharmacy (≥10); (d) oxygen saturation at baseline; (e) pneumonia, confirmed by chest X-ray performed at admission; (f) inflammation, according to C-reactive protein (CRP) and procalcitonin values; and (g) length of stay. These variables were selected after a detailed review of the literature and according to the findings of a prior study of our group [13].

### 2.4. Statistical Analysis

Continuous and categorical variables are expressed as means ± standard deviations and absolute numbers (%), respectively. Data distribution was tested using the Kolmogorov–Smirnov Test. One-way analysis of variance (ANOVA) was conducted to evaluate changes in functional and frailty status across the different assessment points. A longitudinal visualization of frailty and functional trajectories was created using Sankey diagrams using the Flourish Studio (CANVA^®^, London, UK). To assess changes in functional and frailty status over time, the McNemar–Bowker test for paired multinomial data was performed. This test was used to compare transitions between baseline and 1 month, 1 and 12 months, and 12 and 18 months.

Multinomial regressions were performed to examine the associations between candidate risk factors and study outcomes. The “no change” group—those who did not exhibit variations of ≥5 points in the BI and ≥1 in the FAC and FRAIL scales—was used as the reference category. Analyses were adjusted for age and sex (model 1); model 1 + comorbidities, according to the CIRS-G (model 2); model 2 + CRP (model 3); and model 3 + polypharmacy (model 4). Results are shown as relative risk ratios (RRR) and their respective 95% confidence intervals (95% CI). The level of significance was set at *p*-value ≤ 0.05. All analyses were performed with STATA (version 14.0; Stata Corp., College Station, TX, USA).

## 3. Results

### 3.1. Study Flowchart

**Figure 1:**
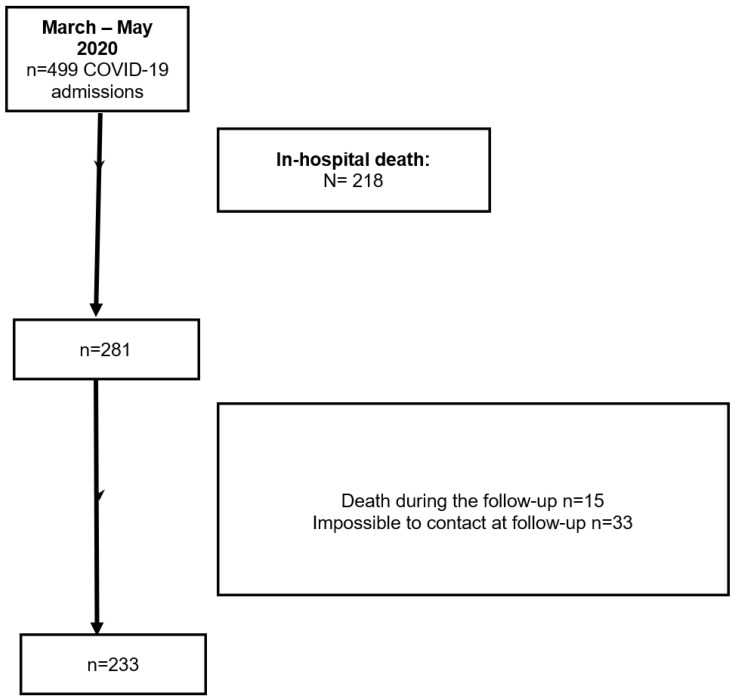
Flow chart of the participants included in the study.

The flowchart of the present study is shown in Figure 1. Two hundred eighty-one older adults were enrolled in the study. Fifteen participants died, and thirty-three did not answer telephone calls during the follow-up. Then, data of 233 participants were examined. No significant differences for the variables examined were observed between included and non-included participants.

### 3.2. Participant Characteristics

The main characteristics of the study participants are shown in Table 1. Average participant age (86.2 ± 4.0) confirms that the examined sample refers to very old adults. Most participants were women (51.1%) and community dwellers (86.3%). The mean length of stay was 15.2 ± 0.7 days. Average BI results (85 ± 1.4) indicate that participants had a mild dependency at admission, with a conserved mobility status, according to FAC scores (4.2 ± 0.1). The majority of participants were prefrail (59.7%), while some were robust (23.6%), and a few were frail (16.7%). The most prevalent conditions were hypertension (80.3%), heart failure (27.9%), and diabetes (27%). Polypharmacy and hyperpolypharmacy were observed in 39.5% and 24% of the sample, respectively. In total, 40.3% of the patients had unilateral pneumonia at their hospital admission, while 43.8% presented bilateral pneumonia.

### 3.3. Functional Trajectories

**Figure 2:**
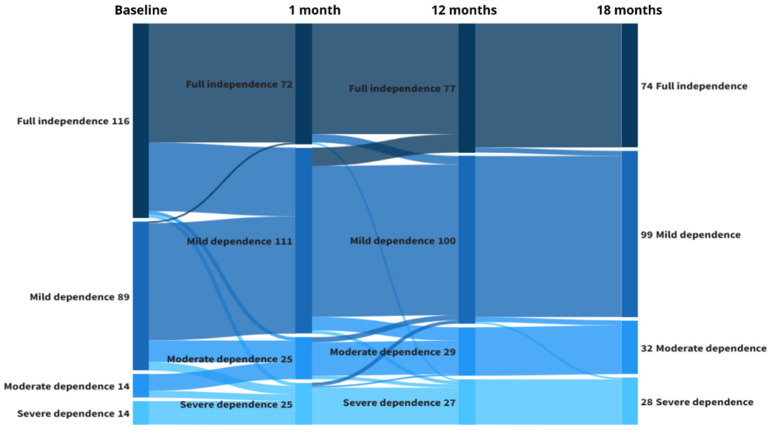
Transitions between dependency levels according to the Barthel Index across the study follow-up. This Sankey diagram illustrates the longitudinal changes in functional status from baseline to 1, 12, and 18 months, as measured using the Barthel Index. Each color represents a dependence category (dark blue for full independence, medium blue for mild dependence, and light blue for moderate to severe dependence). The width of the bands is proportional to the number of participants transitioning between states. The figure highlights both improvement and deterioration in functional independence over time, demonstrating the dynamic nature of disability status during follow-up.

Changes in BI and FAC results over the follow-up are shown in Table 2. Significant decreases in both BI and FAC scores were observed across the follow-up in comparison to baseline. Functional trajectories across the follow-up are shown in Table 3 and Figure 2. No changes (i.e., same score at baseline and follow-up), improvement (i.e., better results at follow-up), and worsening (i.e., detriments at follow-up) patterns of changes were observed. Most participants exhibited worsening in their functional condition. Specifically, nearly 50% suffered significant decreases in BI after 1 month, while 33.4% had reduction in FAC. These rates of impairment were virtually consistent throughout the entire follow-up. Significant changes in functional dependency categories were observed over time, with a pronounced shift occurring between baseline and 1 month (χ^2^ = 68.10, df = 9, *p* < 0.001). In addition, substantial asymmetries in paired flows were observed, most notably the large movement from full independence to mild dependence (41 individuals vs. 11 in the opposite direction) and from mild to moderate dependence (13 vs. 3). In contrast, no significant changes were detected between 1 and 12 months (χ^2^ = 5.86, df = 6, *p* = 0.440), and the distribution remained relatively stable between 12 and 18 months (χ^2^ = 8.00, df = 4, *p* = 0.092), reflecting a plateau in functional status beyond the initial follow-up period.

### 3.4. Frailty Trajectories

**Figure 3:**
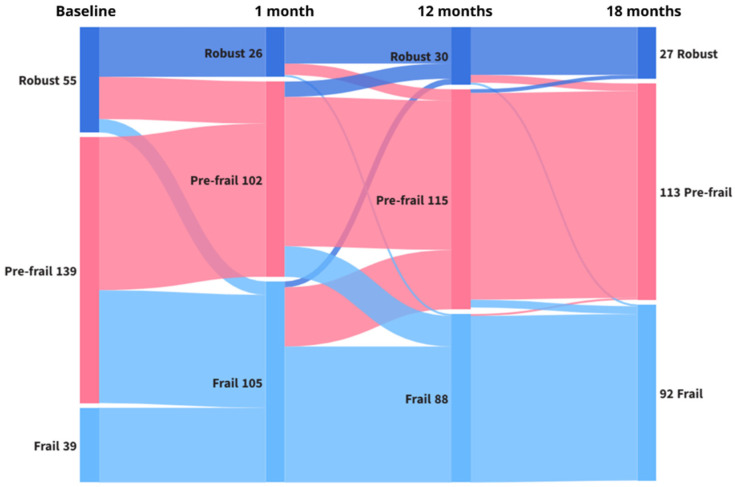
Transitions between frailty states (robust, prefrail, and frail) across the study follow-up. This Sankey diagram illustrates the longitudinal changes in frailty status from baseline to 1, 12, and 18 months. Each color represents a frailty category (blue for robust, pink for prefrail, and light blue for frail), and the width of the bands is proportional to the number of participants transitioning between states. The figure highlights both progression and recovery patterns over time, showing the dynamic nature of frailty status among participants.

Changes on FRAIL results over the follow-up are shown in Table 2. Significant impairments in FRAIL scores were observed across the follow-up period in comparison to baseline. Frailty trajectories across the follow-up are shown in Table 3 and illustrated in Figure 3. After 1 month of discharge, 6 in 10 participants exhibited worsening in frailty status. This value was virtually and slightly reduced in the following evaluation points, but was maintained nearly to 50%. Significant changes in frailty status were observed over time, with a marked shift in category distribution from baseline to 1 month (χ^2^ = 88.0, df = 3, *p* < 0.001), particularly the flows from robust to prefrail (22 vs. 0 in the opposite direction), robust to frail (7 vs. 0), and prefrail to frail (59 vs. 0). No significant changes were detected between 1 and 12 months (χ^2^ = 6.07, df = 3, *p* = 0.108) or between 12 and 18 months (χ^2^ = 3.47, df = 3, *p* = 0.325).

### 3.5. Assessment of Risk Factors

Results of multinomial regressions are shown in Table 4. In the fully adjusted model (model 4), age, length of stay, baseline BI (except in the 1 month after discharge), and frailty status were all independently and significantly associated with worsening BI. Among these risk factors, frailty status showed the highest RRR values.

For worsening in FAC results, significant and independent risk factors were age, FAC scores at baseline, and PCR values (only for the first month). Among these risk factors, baseline FAC punctuation exhibited the highest RRR values.

For worsening in frailty status, the only independent and significant risk factor across all follow-up evaluations was FRAIL scores at baseline. FAC at baseline was a significant risk factor after 1 month of discharge, whereas length of stay was a significant risk factor at month 18.

## 4. Discussion

The main findings of the present study indicate that most very old survivors of COVID-19 experience worsening in their functionality and frailty status within 18 months after discharge. Furthermore, significant and independent risk factors for these scenarios were identified. Specifically, age, functional condition at baseline, length of stay, and inflammation were associated with worsening physical functioning, whereas frailty status at baseline was associated with frailty decline, with eventual contributions of functional condition at baseline and length of stay. These findings highlight the predominant role of pre-existing vulnerability, hospital-related deconditioning, and acute inflammatory response in this cohort of very old COVID-19 survivors. Notably, traditional clinical covariates such as comorbidities, pneumonia severity, and hypoxemia did not emerge as independent predictors. This finding reinforces the necessity of evaluating frailty and functional performance of older adults due to its close relevancy with clinical prognosis. Consistent with our findings, previous studies on in-hospital mortality and long-term outcomes in older COVID-19 patients have similarly identified functional impairment and frailty as the strongest predictors, while comorbidities and markers of acute severity play a marginal role [25].

To the best of our knowledge, this is the first study that examined the long-term effects of COVID-19 infection on physical function and frailty status among very old adults. However, the results of the present investigation align with prior studies focused on younger populations and shorter follow-up periods. Prampart et al. [7] found worsening in frailty status after 3 months of discharge in a younger group of older adults. A study conducted in Japan [16] reported a comparable rate of decline in functionality (54%) within the first year post-hospitalization. A Spanish investigation noted that 42% of the study participants experienced worsening in functional status after 6 months of being discharged after COVID-19 [9].

These findings underscore the critical need for ongoing assessment of physical function and frailty in this population to mitigate the occurrence of adverse events. The progression of disability and frailty increases the risk of falls [26], institutionalization [27], hospitalization [28], and death [29,30], among other complications [27]. Therefore, effective therapies to prevent the worsening of these conditions are essential. To this end, identifying specific risk factors associated with the decline in physical function and frailty is crucial for developing tailored strategies.

Older individuals, who were more dependent to perform the ADLs at admission and had higher CRP values, were at higher risk of experiencing functional decline. These risk factors have also demonstrated significance in other studies examining this theme [16]. Age is a key prognosis marker in COVID-19 patients [30], with other investigations reporting a strong association with mortality in this population [30]. This scenario is likely explained by the fact that, with advancing age, individuals show a reduced capacity to respond to stressors due to significant declines in physiological, cognitive, psychological, and/or social reserves [31,32,33,34]. The importance of this condition is underscored by numerous efforts to develop assessment methods aimed at capturing it (e.g., frailty, intrinsic capacity, biological age) [35,36,37].

The observations that worsened physical condition at baseline was significantly associated with more severe deterioration at follow-up is not surprising, as these results have been repeatedly found in different contexts [38,39,40]. In older adults [40], worsening functionality is more commonly noted among those who already have some degree of impairment, compared to the onset of initial signs in those without symptoms. This decline may reflect the inadequate adoption of healthy lifestyle habits, which prevents the development of disability and mobility issues.

For instance, numerous observational studies have found that physical activity levels are significantly associated with ADL and mobility disability in older adults [40,41]. Boyle et al. [42] investigated the association between physical activity, measured using a self-reported questionnaire, and ADL disability among residents of senior housing facilities. The authors found that each additional hour of physical activity reduced disability risk by 7% [42]. In a hospital setting, low time spent with physical activity and few step counts were significantly associated with hospital-acquired disability. A meta-analysis [41] that analyzed 13 studies and examined more than twenty-five thousand participants revealed that moderate and high physical activity levels significantly reduce the risk of disability in older adults. Moreover, the authors found a higher risk of worsening disability among those with lower physical activity levels [41].

Since the seminal article by Prof. Franceschi et al. [43], which described the potential impact of low-grade inflammation as a key determinant of the aging process, this topic has become a prominent focus in geriatrics and medical sciences. High concentrations of inflammatory markers have long been associated with reduced functionality in older adults [44,45,46]. Specifically, interleukin-6 (IL-6), C-reactive protein (CRP), and tumor necrosis factor-alpha (TNF-α) showed robust cross-sectional associations with frailty status and functional decline in older adults [47]. In the context of post-COVID-19 recovery, elevated pro-inflammatory cytokines during acute infection or persistent low-grade inflammation (inflammaging) may contribute to increased COVID-19 severity, progression to frailty, and physical impairment [48,49,50]. A possible explanation for this relationship is grounded in the fact that inflammation promotes changes at biological levels that predisposes the development of sarcopenia [51,52].

Frailty status at admission was identified as a major determinant of worsening frailty at follow-up. These findings reflect the complexity of frailty and emphasize the severity and clinical consequences of its physiopathology. Similar to the observations with functionality, worsening frailty might be a product of numerous risk factors, including low physical activity [53] and malnutrition [54], which also contributes to its progression. More studies are necessary to provide a more detailed examination of these possible determinants.

Our findings have important clinical implications. We observed that modifiable risk factors, including functional condition, length of stay, inflammation, and frailty status, were significantly associated with worsening events during follow-up. A key initial step is the systematic implementation of comprehensive geriatric assessment (CGA) upon admission to identify those older adults at highest risk of functional decline. The results of this evaluation should be combined with frailty assessment and the evaluation of the presence of inflammation. This early identification allows for the prompt initiation of tailored interventions during and after hospitalization.

Specifically, rehabilitation programs grounded on exercise training protocols are effective to maintain and even improve functional and frailty status during and after hospitalization [55,56,57,58]. These programs have been shown to improve aerobic capacity, muscle strength and mass, physical performance, and independence in activities of daily living, thereby attenuating the impact of baseline deficits, reducing deconditioning, and promoting better long-term recovery. Regarding inflammation, although randomized clinical trials examining the effects of non-pharmacological interventions are often limited to community settings [59,60], it is possible that these strategies might also been employed during hospitalization to reduce inflammatory burden. Changes in length of stay are multifactorial and involve a complicated relationship among numerous variables [61]. Notably, most of the factors examined in this matter involve in-hospital parameters, and just a few studies have been dedicated to describing the associations with pre-hospitalization items [61]. A detailed exploration of each case might be necessary to understand how to reduce hospitalization length.

Hence, strategies to counteract COVID-19 and likely other acute viral respiratory infections might combine two or more of the mentioned approaches to reduce the presence of modifiable factors and thereby the occurrence and/or worsening of functional decline and frailty. Health professionals responsible for older adults’ care need to address the specificities of each patient and understand the most appropriate intervention that can be offered.

The present study has many limitations that need to be acknowledged to provide a better interpretation of our results. First, outcome measures were grounded in self-reported assessment tools, and no performance-based evaluations were conducted to confirm participants’ reports. Hence, the presence of recall bias cannot be ruled out. Second, follow-up visits were conducted through telephone interviews to reduce the risk of contagious. Although one trained geriatrician conducted all evaluations to reduce the risk of evaluation bias and caregivers could be involved to reduce the risk of recall bias, the possibility that face-to-face evaluations could have provided different results cannot be ruled out. Furthermore, it would have allowed the examination of more health aspects. Third, intermediate data collection was not conducted after 6 months of discharge. Results of such analysis could contribute to a better understanding of participants’ trajectories. Fourth, both physical activity levels and nutritional aspects were not assessed in the present study, despite their significant association with physical functioning and frailty. Fifth, only CRP and procalcitonin were assessed as a marker of inflammation. The evaluation of other inflammatory molecules (e.g., interleukin-6, tumor necrosis factor-α) could have provided a more detailed perspective of this topic. Furthermore, biomarkers linked to other biological domains (e.g., hematological, neuromuscular) have been significantly associated with frailty. These data are concerning, as they indicate that a plethora of candidate molecules might be explored in the light of our results [51]. Future studies combining laboratory biomarkers with functional scales are needed to provide a better understanding of this scenario. Sixth, intermediate conditions, such as sarcopenia and malnutrition, were not assessed. Seventh, conducting age-stratified subgroup analyses enhances the clinical applicability of our results. However, our limited sample size at advanced ages for some of the follow-up evaluations (e.g., 51) hampers our capacity to perform such analysis. The use of this statistical approach is even harder given the list of adjusted covariates used.

## 5. Conclusions

Findings of the present study indicate that most very old survivors of COVID-19 experience worsening in their functionality and frailty status within 18 months after discharge. Furthermore, significant and independent risk factors for these scenarios were identified. Specifically, age, functional condition at baseline, length of stay, and inflammation were associated with worsening physical functioning, whereas frailty status at baseline was associated with frailty decline, with eventual contributions of functional condition at baseline and length of stay. Accordingly, interventions aimed at reducing COVID-19 burden should involve the management of these risk factors during hospitalization and within the first periods after discharge.

## Figures and Tables

**Table 1:** Sociodemographic factors, functional status, and chronic morbidities at baseline in old patients discharged after COVID-19 (n = 233).

**Sociodemographic variables**	
Age; mean (SD)	86.2 (4.0)
Male; n (%)	114 (48.9)
Length stay; mean (SD)	15.2 (0.7)
Living; n (%)	
Community dwelling	201 (86.3)
Nursing home	32 (13.7)
**Functional status variables**	
Barthel Index; mean (SD)	85.0 (1.4)
Lawton and Brody Index; mean (SD)	4.9 (0.4)
Functional Ambulatory Classification; mean (SD)	4.2 (0.1)
FRAIL scale; mean (SD)	1.4 (0.1)
**Comorbidities**	
Hypertension; n (%)	187 (80.3)
Diabetes; n (%)	63 (27.0)
Chronic obstructive respiratory disease; n (%)	38 (16.3)
Tuberculosis; n (%)	6 (2.3)
Heart failure; n (%)	65 (27.9)
Ischemic cardiopathy; n (%)	32 (13.7)
Dementia; n (%)	41 (17.6)
Chronic renal failure; n (%)	46 (19.7)
Cerebrovascular disease; n (%)	18 (7.7)
Cancer; n (%)	49 (21.0)
CIRSG score; mean (SD)	10.4 (0.3)
Charlson score; mean (SD)	5.7 (0.1)
**Polypharmacy; n (%)**	
No (<5)	85 (36.5)
Yes (5–9)	92 (39.5)
Hyperpolypharmacy (≥10)	56 (24.0)
**Clinic, laboratory, and X-ray findings**	
Oxygen levels; mean (SD)	93.3 (0.3)
C-reactive protein; mean (SD)	7.9 (0.4)
Procalcitonin; mean (SD)	0.4 (0.1)
Chest X-ray; n (%)	
No pneumonia	37 (15.9)
Unilateral pneumonia	94 (40.3)
Bilateral pneumonia	102 (43.8)

SD: Standard deviation; %: percentage.

**Table 2:** Mean (SD) of functional variables at baseline, 1 month, 12 months, and 18 months after discharge for COVID-19 (n = 233).

	BASELINE	1 MONTH	*p* Value	12 MONTHS	*p* Value	18 MONTHS	*p* Value
**BARTHEL INDEX**	85.04 ±1.44	76.33 ± 1.75	**<0.001**	75.9 ± 1.79	**<0.001**	75.15 ± 1.83	**<0.001**
**FRAIL SCALE**	1.39 ± 0.08	2.36 ± 0.10	**<0.001**	2.09 ± 0.09	**0.004**	2.16 ± 0.09	**0.002**
**FUNCTIONAL AMBULATORY CLASSIFICATION**	4.24 ± 0.09	3.61 ± 0.11	**<0.001**	3.69 ± 0.11	**<0.001**	3.62 ± 0.11	**<0.001**

*p*-Values for differences in means were obtained using ANOVA or Kruskal–Wallis tests, as appropriate.

**Table 3:** Changes in functional variables at 1 month, 12 months, and 18 months after discharge for COVID-19, compared to baseline situation (n = 233).

	1 MONTH			*p* Value	12 MONTHS			*p* Value	18 MONTHS			*p* Value
	No Changes	Improvement	Worsening		No Changes	Improvement	Worsening		No Changes	Improvement	Worsening	
**BARTHEL INDEX (n, %)**	123 (52.79)	5 (2.15)	105 (45.06)	**<0.001**	120 (51.50)	6 (2.58)	107 (45.92)	**<0.001**	116 (49.79)	6 (2.58)	111 (47.64)	**<0.001**
**FRAIL SCALE** **(n, %)**	94 (40.34)	3 (1.29)	136 (58.37)	**<0.001**	111 (47.64)	6 (2.58)	116 (49.79)	**<0.001**	105 (45.26)	6 (2.58)	121 (52.16)	**<0.001**
**FUNCTIONAL AMBULATORY CLASSIFICATION (n, %)**	152 (65.24)	3 (1.29)	78 (33.48)	**<0.001**	162 (69.53)	2 (0.86)	69 (29.61)	**<0.001**	156 (66.95)	2 (0.86)	75 (32.19)	**<0.001**

*p*-Values for differences were obtained from chi-square tests.

**Table 4:** Factors associated with a decline in the functional situation (according to the Barthel Index, FRAIL score, and Functional Ambulatory Classification) among older adults discharged after COVID-19 disease.

	AGE RRR (95% CI)	GENDER RRR (95% CI)	LENGTH STAY RRR (95% CI)	BI RRR (95% CI)	FRAIL SCORE RRR (95% CI)	FAC RRR (95% CI)	COMORBIDITIES RRR (95% CI)	CRP RRR (95% CI)	PNEUMONIA RRR (95% CI)		OXYGEN LEVELS RRR (95% CI)	POLYPHARMACY RRR (95% CI)
									Unilateral	Bilateral		
**BI worsening**												
1 month (n = 105)	1.09 (1.02–1.17) **	0.84 (0.49–1.44)	1.03 (1.00–1.07) *	1.01 (0.98–1.02)	1.43 (1.12–1.82) **	0.82 (0.67–1.02)	0.85 (0.65–1.12)	1.01 (0.97–1.05)	1.03 (0.46–2.30)	0.88 (0.40–1.91)	0.99 (0.94–1.06)	1.51 (0.93–2.46)
12 months (n = 107)	1.12 (1.04–1.20) **	0.76 (0.44–1.31)	1.03 (1.00–1.07) *	1.01 (1.00–1.03) *	1.38 (1.09–1.75) **	0.84 (0.68–1.04)	0.83 (0.64–1.09)	1.00 (0.95–1.04)	1.07 (0.48–2.42)	0.85 (0.39–1.85)	1.02 (0.97–1.09)	0.97 (0.60–1.58)
18 months (n = 111)	1.11 (1.04–1.19) **	0.77 (0.44–1.32)	1.03 (1.00–1.06) *	1.01 (1.00–1.03) *	1.37 (1.08–1.74) **	0.84 (0.68–1.03)	0.85 (0.65–1.11)	0.99 (0.95–1.04)	1.02 (0.45–2.29)	0.83 (0.38–1.82)	1.03 (0.97–1.09)	0.99 (0.61–1.60)
**FRAIL worsening**												
1 month (n = 136)	1.01 (0.94–1.08)	0.72 (0.42–1.24)	1.02 (0.99–1.04)	0.99 (0.97–1.02)	1.32 (1.04–1.69) *	1.24 (1.01–1.52) *	1.10 (0.84–1.43)	0.99 (0.96–1.04)	1.10 (0.49–2.48)	0.92 (0.42–1.99)	1.01 (0.95–1.07)	1.06 (0.66–1.70)
12 months (n = 116)	1.03 (0.96–1.10)	0.79 (0.46–1.36)	1.03 (0.99–1.06)	1.00 (0.98–1.01)	1.29 (1.01–1.66) *	1.21 (0.98–1.49)	1.29 (0.98–1.68)	0.97 (0.93–1.02)	1.64 (0.73–3.69)	1.04 (0.48–2.26)	1.02 (0.96–1.08)	1.07 (0.66–1.70)
18 months (n = 121)	1.06 (0.99–1.13)	0.65 (0.38–1.11)	1.03 (1.00–1.06) *	1.00 (0.98–1.01)	1.39 (1.08–1.80) **	1.17 (0.95–1.44)	1.18 (0.90–1.54)	0.98 (0.94–1.03)	1.89 (0.83–4.29)	1.33 (0.61–2.91)	1.03 (0.97–1.09)	1.02 (0.64–1.64)
**FAC worsening**												
1 month (n = 78)	1.14 (1.06–1.23) ***	0.86 (0.48–1.54)	1.01 (0.98–1.04)	1.00 (0.99–1.01)	0.94 (0.74–1.20)	1.36 (1.04–1.77) *	0.94 (0.71–1.25)	1.05 (1.00–1.10) *	1.10 (0.46–2.64)	0.97 (0.41–2.29)	1.00 (0.94–1.07)	1.10 (0.66–1.83)
12 months (n = 69)	1.16 (1.07–1.24) ***	0.65 (0.35–1.20)	1.02 (0.99–1.05)	1.00 (0.99–1.02)	1.01 (0.79–1.31)	1.45 (1.09–1.94) **	1.20 (0.89–1.61)	1.03 (0.98–1.08)	1.01 (0.42–2.47)	0.65 (0.27–1.58)	1.01 (0.95–1.08)	0.74 (0.43–1.26)
18 months (n = 75)	1.15 (1.07–1.23) ***	0.69 (0.38–1.25)	1.01 (0.98–1.04)	1.00 (0.99–1.02)	1.05 (0.82–1.34)	1.51 (1.13–2.02) **	1.30 (0.98–1.75)	1.04 (0.99–1.09)	1.25 (0.52–3.00)	0.77 (0.32–1.84)	1.02 (0.96–1.09)	0.92 (0.55–1.54)

BI: Barthel Index; CI: confidence interval; CRP: C-reactive protein; FAC: Functional Ambulatory Classification; RRR: relative risk ratio. All models were adjusted for baseline age, sex, comorbidities according to CIRS-G score, C-reactive protein, and polypharmacy. Reference category: no changes in functional status according to Barthel Index, FRAIL score, or FAC. * *p* < 0.05, ** *p* < 0.01, *** *p* < 0.001.

## Data Availability

The original contributions presented in this study are included in the article. Further inquiries can be directed to the corresponding authors.

## References

[1] COVID-19 Cases|WHO COVID-19 Dashboard. https://data.who.int/dashboards/covid19/cases.

[2] STARSurg Collaborative and COVIDSurg Collaborative (2021). Death following pulmonary complications of surgery before and during the SARS-CoV-2 pandemic. Br. J. Surg..

[3] Wu J.T., Leung K., Bushman M., Kishore N., Niehus R., de Salazar P.M., Cowling B.J., Lipsitch M., Leung G.M. (2020). Estimating Clinical Severity of COVID-19 from the Transmission Dynamics in Wuhan, China. Nat. Med..

[4] Ramos-Rincon J.M., Buonaiuto V., Ricci M., Martín-Carmona J., Paredes-Ruíz D., Calderón-Moreno M., Rubio-Rivas M., Beato-Pérez J.L., Arnalich-Fernández F., Monge-Monge D. (2021). Clinical Characteristics and Risk Factors for Mortality in Very Old Patients Hospitalized with COVID-19 in Spain. J. Gerontol. Ser. A.

[5] Seligman B., Wysham K.D., Shahoumian T., Orkaby A.R., Goetz M.B., Osborne T.F., Smith V.A., Maciejewski M.L., Hynes D.M., Boyko E.J. (2024). Change in Frailty among Older COVID-19 Survivors. J. Am. Geriatr. Soc..

[6] Pérez-Rodríguez P., Díaz de Bustamante M., Aparicio Mollá S., Arenas M.C., Jiménez-Armero S., Lacosta Esclapez P., González-Espinoza L., Bermejo Boixareu C. (2021). Functional, Cognitive, and Nutritional Decline in 435 Elderly Nursing Home Residents after the First Wave of the COVID-19 Pandemic. Eur. Geriatr. Med..

[7] Prampart S., Le Gentil S., Bureau M.L., Macchi C., Leroux C., Chapelet G., de Decker L., Rouaud A., Boureau A.S. (2022). Functional Decline, Long Term Symptoms and Course of Frailty at 3-Months Follow-up in COVID-19 Older Survivors, a Prospective Observational Cohort Study. BMC Geriatr..

[8] Andrew M.K., MacDonald S., Godin J., McElhaney J.E., LeBlanc J., Hatchette T.F., Bowie W., Katz K., McGeer A., Semret M. (2021). Persistent Functional Decline Following Hospitalization with Influenza or Acute Respiratory Illness. J. Am. Geriatr. Soc..

[9] Carrillo-Garcia P., Garmendia-Prieto B., Cristofori G., Lozano-Montoya I., Gómez-Pavón J. (2022). Health Impact on the Elderly Survivors of COVID-19: Six Months Follow Up. Rev. Esp. Geriatr. Gerontol..

[10] van Seben R., Covinsky K.E., Reichardt L.A., Aarden J.J., van der Schaaf M., van der Esch M., Engelbert R.H.H., Twisk J.W.R., Bosch J.A., Buurman B.M. (2020). Insight Into the Posthospital Syndrome: A 3-Month Longitudinal Follow up on Geriatric Syndromes and Their Association with Functional Decline, Readmission, and Mortality. J. Gerontol. Ser. A.

[11] Neumann-Podczaska A., Al-Saad S.R., Karbowski L.M., Chojnicki M., Tobis S., Wieczorowska-Tobis K. (2020). COVID 19—Clinical Picture in the Elderly Population: A Qualitative Systematic Review. Aging Dis..

[12] Chen Y.T., Shao S.C., Lai E.C.C., Hung M.J., Chen Y.C. (2020). Mortality Rate of Acute Kidney Injury in SARS, MERS, and COVID-19 Infection: A Systematic Review and Meta-Analysis. Crit. Care.

[13] Rodríguez-Sánchez I., Redondo-Martín M., Furones-Fernández L., Méndez-Hinojosa M., Chen-Chim Á., Saavedra-Palacios R., Gil-Gregorio P. (2021). Functional, Clinical, and Sociodemographic Variables Associated with Risk of In-Hospital Mortality by COVID-19 in People over 80 Years Old. J. Nutr. Health Aging.

[14] López Izquierdo R., Ruiz Albi T., Bermejo Martín J.F., Almansa Mora R., Villafañe Sanz F.V., Arroyo Olmedo L., Urbina Carrera C.A., Sánchez Ramón S., Martín Rodríguez F., Moreno Torrero F. (2021). Modelos de Riesgo Para La Predicción de Mortalidad Hospitalaria En Ancianos Con Neumonía Por COVID-19. Emerg. Rev. Cient. Soc. Esp. Med. Urgenc. Emerg..

[15] Seong G.M., Baek A.R., Baek M.S., Kim W.Y., Kim J.H., Lee B.Y., Na Y.S., Lee S.I. (2021). Comparison of Clinical Characteristics and Outcomes of Younger and Elderly Patients with Severe COVID-19 in Korea: A Retrospective Multicenter Study. J. Pers. Med..

[16] Hosoda T., Hamada S. (2021). Functional Decline in Hospitalized Older Patients with Coronavirus Disease 2019: A Retrospective Cohort Study. BMC Geriatr..

[17] Izaguirre P., Arakaki É., Boero J.V., Zalazar Á., Ghirlanda M., Caruso D. (2023). Functional Status in Older Adults Following Hospitalization for COVID-19: A Cohort Study. Ann. Geriatr. Med. Res..

[18] Dias M.B., Avelino-Silva T.J., Ferriolli E., Taniguchi L.U., Jacob-Filho W., Suemoto C.K., Aliberti M.J.R. (2025). Post-Discharge COVID-19 Symptoms Predict 1-Year Functional Decline, Falls, and Emergency Department Visits: A Cohort Study. J. Am. Geriatr. Soc..

[19] Cuschieri S. (2019). The STROBE Guidelines. Saudi J. Anaesth..

[20] Mahoney F.I., Barthel D.W. (1965). Functional Evaluation: The Barthel Index. Md. State Med. J..

[21] Abellan Van Kan G., Rolland Y., Bergman H., Morley J.E., Kritchevsky S.B., Vellas B. (2008). The I.A.N.A. Task Force on Frailty Assessment of Older People in Clinical Practice. J. Nutr. Health Aging.

[22] Holden M.K., Gill K.M., Magliozzi M.R., Nathan J., Piehl-Baker L. (1984). Clinical Gait Assessment in the Neurologically Impaired: Reliability and Meaningfulness. Phys. Ther..

[23] Charlson M.E., Pompei P., Ales K.L., MacKenzie C.R. (1987). A New Method of Classifying Prognostic Comorbidity in Longitudinal Studies: Development and Validation. J. Chronic Dis..

[24] Miller M.D., Paradis C.F., Houck P.R., Mazumdar S., Stack J.A., Rifai A.H., Mulsant B., Reynolds C.F. (1992). Rating Chronic Medical Illness Burden in Geropsychiatric Practice and Research: Application of the Cumulative Illness Rating Scale. Psychiatry Res..

[25] Laosa O., Pedraza L., Álvarez-Bustos A., Carnicero J.A., Rodriguez-Artalejo F., Rodriguez-Mañas L. (2020). Rapid Assessment at Hospital Admission of Mortality Risk from COVID-19: The Role of Functional Status. J. Am. Med. Dir. Assoc..

[26] Tejiram S., Cartwright J., Taylor S.L., Hatcher V.H., Galet C., Skeete D.A., Romanowski K.S. (2021). A Prospective Comparison of Frailty Scores and Fall Prediction in Acutely Injured Older Adults. J. Surg. Res..

[27] Yu M., Ding J., Wu X., Wen X., Jin J., Wang H., Lv D., Zhao S., Jiao J., Xu T. (2025). Indication of Frailty Transitions on 2-Year Adverse Health Outcomes among Older Chinese Inpatients: Insight from a Multicenter Prospective Cohort Study. PLoS ONE.

[28] Álvarez-Bustos A., Carnicero-Carreño J.A., Sanchez-Sanchez J.L., Garcia-Garcia F.J., Alonso-Bouzón C., Rodríguez-Mañas L. (2022). Associations between Frailty Trajectories and Frailty Status and Adverse Outcomes in Community-Dwelling Older Adults. J. Cachexia Sarcopenia Muscle.

[29] Gill T.M., Gahbauer E.A., Han L., Allore H.G. (2010). Trajectories of Disability in the Last Year of Life. N. Engl. J. Med..

[30] Bonanad C., García-Blas S., Tarazona-Santabalbina F., Sanchis J., Bertomeu-González V., Fácila L., Ariza A., Núñez J., Cordero A. (2020). The Effect of Age on Mortality in Patients with COVID-19: A Meta-Analysis with 611,583 Subjects. J. Am. Med. Dir. Assoc..

[31] Tucker A.M., Stern Y. (2011). Cognitive Reserve in Aging. Curr. Alzheimer Res..

[32] Troen B.R. (2003). The Biology of Aging. Mt. Sinai J. Med..

[33] Ihle A., Oris M., Baeriswyl M., Zuber S., Cullati S., Maurer J., Kliegel M. (2021). The Longitudinal Relation between Social Reserve and Smaller Subsequent Decline in Executive Functioning in Old Age Is Mediated via Cognitive Reserve. Int. Psychogeriatr..

[34] Stern Y., Albert M., Barnes C.A., Cabeza R., Pascual-Leone A., Rapp P.R. (2023). A Framework for Concepts of Reserve and Resilience in Aging. Neurobiol. Aging.

[35] Fried L.P., Tangen C.M., Walston J., Newman A.B., Hirsch C., Gottdiener J., Seeman T., Tracy R., Kop W.J., Burke G. (2001). Frailty in Older Adults: Evidence for a Phenotype. J. Gerontol. Ser. A.

[36] Bautmans I., Knoop V., Amuthavalli Thiyagarajan J., Maier A.B., Beard J.R., Freiberger E., Belsky D., Aubertin-Leheudre M., Mikton C., Cesari M. (2022). WHO Working Definition of Vitality Capacity for Healthy Longevity Monitoring. Lancet Healthy Longev..

[37] Mathur A., Taurin S., Alshammary S. (2024). New Insights into Methods to Measure Biological Age: A Literature Review. Front. Aging.

[38] Koch M.W., Mostert J., Repovic P., Bowen J.D., Wolinsky J.S., Lublin F.D., Strijbis E., Cutter G. (2022). Early First-line Treatment Response and Subsequent Disability Worsening in Relapsing–Remitting Multiple Sclerosis. Eur. J. Neurol..

[39] Abdelhak A., Antweiler K., Kowarik M.C., Senel M., Havla J., Zettl U.K., Kleiter I., Hoshi M.-M., Skripuletz T., Haarmann A. (2024). Patient-Reported Outcome Parameters and Disability Worsening in Progressive Multiple Sclerosis. Mult. Scler. Relat. Disord..

[40] Balzi D., Lauretani F., Barchielli A., Ferrucci L., Bandinelli S., Buiatti E., Milaneschi Y., Guralnik J.M. (2010). Risk Factors for Disability in Older Persons over 3-Year Follow-Up. Age Ageing.

[41] Tak E., Kuiper R., Chorus A., Hopman-Rock M. (2013). Prevention of Onset and Progression of Basic ADL Disability by Physical Activity in Community Dwelling Older Adults: A Meta-Analysis. Ageing Res. Rev..

[42] Boyle P.A., Buchman A.S., Wilson R.S., Bienias J.L., Bennett D.A. (2007). Physical Activity Is Associated with Incident Disability in Community-Based Older Persons. J. Am. Geriatr. Soc..

[43] Franceschi C., Bonafè M., Valensin S., Olivieri F., De Luca M., Ottaviani E., De Benedictis G. (2000). Inflamm-aging: An Evolutionary Perspective on Immunosenescence. Ann. N. Y. Acad. Sci..

[44] Cesari M., Penninx B.W.J.H., Pahor M., Lauretani F., Corsi A.M., Williams G.R., Guralnik J.M., Ferrucci L. (2004). Inflammatory Markers and Physical Performance in Older Persons: The InCHIANTI Study. J. Gerontol. Ser. A.

[45] Verghese J., Holtzer R., Lipton R.B., Wang C. (2012). High-Sensitivity C-Reactive Protein and Mobility Disability in Older Adults. Age Ageing.

[46] Penninx B.W.J.H., Kritchevsky S.B., Newman A.B., Nicklas B.J., Simonsick E.M., Rubin S., Nevitt M., Visser M., Harris T., Pahor M. (2004). Inflammatory Markers and Incident Mobility Limitation in the Elderly. J. Am. Geriatr. Soc..

[47] Xu Y.S., Wang M.M., Chen D., Jiang X., Xiong Z.F. (2022). Inflammatory Biomarkers in Older Adults with Frailty: A Systematic Review and Meta-Analysis of Cross-Sectional Studies. Aging Clin. Exp. Res..

[48] Wanhella K.J., Fernandez-Patron C. (2022). Biomarkers of Ageing and Frailty May Predict COVID-19 Severity. Ageing Res. Rev..

[49] Knopp P., Miles A., Webb T.E., Mcloughlin B.C., Mannan I., Raja N., Wan B., Davis D. (2020). Presenting Features of COVID-19 in Older People: Relationships with Frailty, Inflammation and Mortality. Eur. Geriatr. Med..

[50] El Assar M., Rodríguez-Sánchez I., Álvarez-Bustos A., Rodríguez-Mañas L. (2024). Biomarkers of Frailty. Mol. Asp. Med..

[51] Picca A., Coelho-Junior H.J., Calvani R., Marzetti E., Vetrano D.L. (2022). Biomarkers Shared by Frailty and Sarcopenia in Older Adults: A Systematic Review and Meta-Analysis. Ageing Res. Rev..

[52] Coelho-Junior H.J., Marzetti E., Sexton C.L., Wu K., Mankowski R., Anton S.D., Leeuwenburgh C., Picca A. (2024). Mitochondrial Quality Control Measures, Systemic Inflammation, and Lower-Limb Muscle Power in Older Adults: A PROMPT Secondary Analysis. J. Nutr. Health Aging.

[53] Zhao W., Hu P., Sun W., Wu W., Zhang J., Deng H., Huang J., Ukawa S., Lu J., Tamakoshi A. (2022). Effect of Physical Activity on the Risk of Frailty: A Systematic Review and Meta-Analysis. PLoS ONE.

[54] Sella-Weiss O. (2021). Association between Swallowing Function, Malnutrition and Frailty in Community Dwelling Older People. Clin. Nutr. ESPEN.

[55] Araya-Quintanilla F., Sepulveda-Loyola W., Cuyul-Vásquez I., Alvarez-Bustos A., Gutiérrez-Espinoza H., Suziane Probst V., Camp P.G., Rodríguez-Mañas L. (2023). Recommendations and Effects of Rehabilitation Programs in Older Adults After Hospitalization for COVID-19: A Scoping Review. Am. J. Phys. Med. Rehabil..

[56] Martínez-Velilla N., Casas-Herrero A., Zambom-Ferraresi F., Sáez de Asteasu M.L., Lucia A., Galbete A., García-Baztán A., Alonso-Renedo J., González-Glaría B., Gonzalo-Lázaro M. (2019). Effect of Exercise Intervention on Functional Decline in Very Elderly Patients During Acute Hospitalization: A Randomized Clinical Trial. JAMA Intern. Med..

[57] Carneiro M.A.S., Franco C.M.C., Silva A.L., Castro-E-Souza P., Kunevaliki G., Izquierdo M., Cyrino E.S., Padilha C.S. (2021). Resistance exercise intervention on muscular strength and power, and functional capacity in acute hospitalized older adults: A systematic review and meta-analysis of 2498 patients in 7 randomized clinical trials. GeroScience.

[58] Shivgulam M.E., Liu H., Zafar Z., Cheema M., Ahmadi S., Theou O., O’Brien M.W. (2026). In-hospital exercise interventions improve inpatients’ frailty and demonstrate promising implementation outcomes: A systematic review and meta-analysis. Ageing Res. Rev..

[59] Petersen A.M., Pedersen B.K. (2005). The anti-inflammatory effect of exercise. J. Appl. Physiol..

[60] Martínez-González M.A., Salas-Salvadó J., Estruch R., Corella D., Fitó M., Ros E., PREDIMED INVESTIGATORS (2015). Benefits of the Mediterranean Diet: Insights from the PREDIMED Study. Prog. Cardiovasc. Dis..

[61] Stone K., Zwiggelaar R., Jones P., Mac Parthaláin N. (2022). A systematic review of the prediction of hospital length of stay: Towards a unified framework. PLoS Digit. Health.

